# Building capacity for Public Health 3.0: introducing implementation science into an MPH curriculum

**DOI:** 10.1186/s13012-019-0866-6

**Published:** 2019-02-28

**Authors:** Rohit Ramaswamy, Joe Mosnier, Kristin Reed, Byron J. Powell, Anna P. Schenck

**Affiliations:** Gillings School of Global Public Health, University of North Carolina, Chapel Hill, USA

**Keywords:** Implementation science, Global health, Capacity building, Public Health 3.0

## Abstract

**Background:**

Many public health programs fail because of an inability to implement tested interventions in diverse, complex settings. The field of implementation science is engaged in developing strategies for successful implementation, but current training is primarily researcher-focused. To tackle the challenges of the twenty-first century, public health leaders are promoting a new model titled Public Health 3.0 where public health practitioners become “chief health strategists” and develop interdisciplinary skills for multisector engagement to achieve impact. This requires broad training for public health practitioners in implementation science that includes the allied fields of systems and design thinking, quality improvement, and innovative evaluation methods. At UNC Chapel Hill’s Gillings School of Global Public Health, we created an interdisciplinary set of courses in applied implementation science for Master of Public Health (MPH) students and public health practitioners. We describe our rationale, conceptual approach, pedagogy, courses, and initial results to assist other schools contemplating similar programs.

**Methods:**

Our conceptual approach recognized the vital relationship between implementation research and practice. We conducted a literature review of thought leaders in public health to identify skill areas related to implementation science that are priorities for the future workforce. We also reviewed currently available training programs in implementation science to understand their scope and objectives and to assess whether any of these would be a fit for these priorities. We used a design focused implementation framework to create four linked courses drawing from multiple fields such as engineering, management, and the social sciences and emphasizing application through case studies. We validated the course content by mapping them to implementation science competencies in the literature.

**Results:**

To date, there is no other program that provides comprehensive interdisciplinary skills in applied implementation science for MPH students. As of April 2018, we have offered a total of eleven sections of the four courses, with a total enrollment of 142, of whom 127 have been master’s-level students in the school of public health. Using Kirkpatrick’s Model, we found positive student reaction, learning, and behavior. Many students have completed applied implementation science focused practicums, master’s papers, and special studies.

**Conclusions:**

A systematically designed interdisciplinary curriculum in applied implementation science for MPH students has been found by students to be a useful set of skills. Students have demonstrated the capability to master this material and incorporate it into their practicums and master’s papers.

**Electronic supplementary material:**

The online version of this article (10.1186/s13012-019-0866-6) contains supplementary material, which is available to authorized users.

## Background

### Building skills for the next generation of public health leaders

It is widely recognized that many public health programs fail to achieve their full potential and that emerging trends including climate change, globalization, demographic transitions, and the influence of social media will pose new challenges to public health that require innovative interdisciplinary solutions [1, 2]. In 2016, the U.S. Department of Health and Human Services launched Public Health 3.0, promoting the transformation of public health officials from professional managers of public health agencies to “chief health strategists” leading essential cross-sector collaborations [3]. In 2015, the United Nations launched the Sustainable Development Goals (SDGs), shaped by an unprecedented vision of transformative improvement in health and wellbeing. As several authors have stated, the success of these ambitious endeavors rests on expanded innovation not just in science and medicine, but also in methods for rapid data collection, program design and implementation, and evaluation to be responsive to the challenges of tomorrow. For example, Erwin and Brownson observe that while evidence-based interventions take time and need numerous studies, public health crises such as Ebola and Zika require immediate action despite limited knowledge about the most efficacious interventions [2]*.* Using the example of small pox eradication, Frieden describes how effective public health programs require innovations not just in biological intervention (i.e., the vaccine) but also in surveillance processes, organizational management, and service delivery methods [1].

Therefore, while innovations, broadly defined, are undoubtedly key to success in public health, the evidence of their effectiveness is not confirmed in laboratories, but in their demonstrated implementation in diverse settings and contexts. However, the gap in bringing these innovations to fruitful implementation on a large scale in diverse, complex settings is also well known. Real-world implementation of evidence-based interventions has been described by the World Health Organization as “one of the greatest challenges … [for] the global health community” [4]. The result has been that populations, particularly those in low- and middle-income countries, too often gain little from promising innovations because these innovations never make their way consistently into settings where they can add the most value.

The field of implementation science—“the study of methods for improving the uptake, implementation, and translation of research findings into routine and common practice”—has emerged to help investigate methods to facilitate the real-world adoption in the field of interventions that have been shown to work in research settings [5–7], and its importance in low-income settings is well recognized [8–10]. Therefore, as we enter the era of Public Health 3.0, it has become critical to train public health practitioners and students in a broad set of implementation science skills that enable them to implement, improve, and evaluate global and local public health programs.

### Training needs for implementation science practitioners

**Fig. 1:**
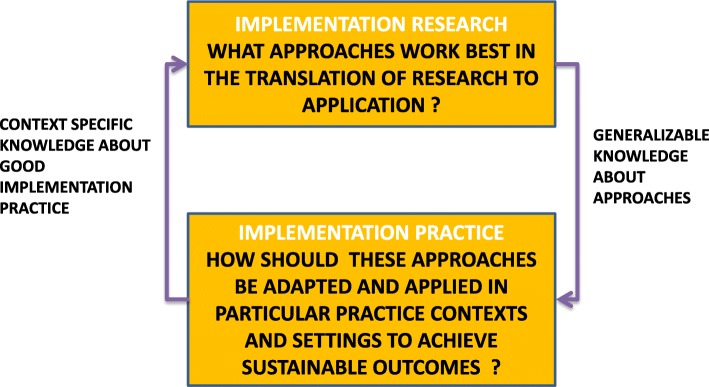
The dual roles of implementation research and practice

Given that the objective of the field is to help translate research findings to practice, programs to teach implementation science must begin by acknowledging the necessary relationship between researchers and practitioners. We conceptualized this by creating a model depicting implementation science as the connection between two equally important components, *implementation research* and *implementation practice*, as shown in Fig. 1.

*Implementation research*, shown in the upper box of Fig. 1, involves scientific inquiry on the questions regarding implementation [11]. Researchers from diverse fields including medicine, nursing, pharmacy, social work, engineering, and management develop theories and frameworks about the mechanisms and processes that create barriers to the uptake of innovations or tested interventions into practice and create and test targeted implementation strategies to address these mechanisms. The unique nature of implementation research requires, by definition, that strategies can only be tested in the space where actual implementation happens. Therefore, to advance scientific inquiry into implementation, implementation research must take place in the everyday world of *implementation practice*, shown in the lower box of Fig. 1. This is the world of practitioners, who are responsible for program implementation, management, or evaluation, described by Kirchner et al. as the agents who promote evidence-based interventions by using implementation tools and through collaboration with implementation experts in order to employ evidence-based strategies [12]. Implementers must systematically and rigorously employ the theory informed frameworks and strategies proposed by the researchers in routine practice in particular settings. This link is suggested by Fig. 1’s downward-facing arrow. Insights from these local implementation efforts, when carefully documented, provide invaluable practice-based evidence about what works, why, and when. This evidence, when disseminated to researchers as shown by the upward-facing arrow on the left of Fig. 1, enables researchers to strengthen generalizable knowledge about what works. This inseparable relationship between implementation researchers and practitioners is emphasized by Peterson et al. who call this the “virtuous cycle where research informs practice and practice informs research” [13]. Training for both researchers and practitioners must enable each group to easily navigate the virtuous cycle of Fig. 1.

However, this does not represent the current state of training in the field. Theories, models, and frameworks have proliferated with limited guidance on how and when they are to be used, resulting in poor or variable implementation practices. As a result, documentation about how practitioners are using the findings from implementation research is poor, inconsistent, and difficult to interpret [14]. Although guidelines for rigorous documentation of the process of implementation in routine practice are now emerging, such documentation is labor-intensive and complete data to properly drive the feedback loop is rarely collected outside of carefully controlled research projects [15]. As a result, ironically, a research-to-practice gap persists in a field invented to close this gap. Geng et al. [16] describe this gap as the tension between “rigor” and “relevance”.

A panel of D&I experts including leaders of current trainings, NIH center directors, and trainees in existing programs convened by NIH in 2016 to develop a field-wide perspective on training for D&I researchers also concluded that training is not readily available for practitioners. The panel stated that while there are academic institutions that provide training for professional and research-focused graduate students, the majority of these programs tend to focus toward postdoctoral and more experienced professionals who come into D&I from related fields. The panel recommended that D&I practice training be available for all people who have a role in implementation [17]. The need for training for implementers also been identified by Park et al. [18] and Moore et al. [19] in papers evaluating a training initiative and a course developed to build implementation practitioner competencies.

In 2015, the Gillings School of Global Public Health at the University of North Carolina (UNC) at Chapel Hill developed an interdisciplinary, four-course sequence in *Applied Implementation Science* as part of a Master of Public Health (MPH) degree program, seeking thereby to provide MPH students with a full complement of practical skills in implementation science.

The objective of this paper is to describe the rationale for the development of the curriculum at UNC, the conceptual framework used, the courses developed, and the pedagogy. We also present student evaluation results and lessons learned. We hope that our experience will be of service to schools of public health contemplating similar programs or to institutions seeking to develop a comprehensive practitioner focused training offering.

## Methods

### Identifying Public Health 3.0 training needs

We reviewed the literature to gather the views of public health leaders about priority areas for training on Public Health 3.0. The objective was not to do an exhaustive search but to explore the opinions of key thought leaders to use as a basis for the curriculum development. We conducted a preliminary search on Google using “Public Health 3.0 practitioner skills” and “Public Health 3.0 practitioner competencies” and followed up with a search through the university library system online catalog. We reviewed the results to identify needed skills related to implementation science and allied fields. Teutsch and Fielding, in their article “Rediscovering the Core of Public Health,” observe that future public health practitioners need skills such as “policy analysis, communication, evaluation and quality improvement” [20]. DeSalvo et al. emphasize the need for building the ability to acquire a systems perspective and act accordingly and to realize and use the power of “new types of data”, not just health outcomes [3]. Erwin and Brownson also emphasize the need for systems thinking and assert that signficant curricular changes will be needed [2].

### Reviewing existing implementation science training programs

We conducted a review of implementation science training programs to map the landscape of what is currently available so that we could determine if any existing programs met the Public Health 3.0 training needs in implementation science. Since descriptions of many training programs are unlikely to be published in the research literature, we also began our initial searches on Google. We used the following terms: “implementation science training programs,” “implementation science courses,” and “graduate programs implementation science,” and repeated the searches by substituting “research” for “science” and adding “dissemination.” We supplemented the Google search by reviewing several online sites that curate aggregated lists of training opportunities [21–26]; a recent published survey of existing dissemination and implementation (D&I) research training programs [27]; a systematic review of US-based D&I training efforts by researchers interested in integrating implementation science training elements into medical school curricula [28]; and by searching for academic papers related to training programs in the journal *Implementation Science*.

### Categorization of training offerings

We categorized the training offerings from our review into three groups: (i) non-academic (i.e., not part of a university degree, diploma, or certificate program) intensive workshops and other short-term courses, including online modular units; (ii) single, stand-alone implementation science courses offered in academic institutions; and (iii) certificate-, master’s-, and PhD-level programs on implementation science offered by schools of medicine and public health.

### Assessing accessibility and fit of current offerings to public health practitioners

To determine the extent to which current programs met these needs or were accessible to MPH students and other public health practitioners, we assessed the programs offering graduate credentials in implementation science based on the following criteria: (a) whether the program is US based; (b) if it is offered by a school of public health; and (c) whether it is targeted toward students pursuing a practice-based degree (rather than a research degree). Non-US-based programs are out of reach for most students and practitioners living in the USA and potentially needing Public Health 3.0 skills. Similarly, public health professionals often do not have access to programs offered in medical schools. Finally, while individual doctoral level courses may be available to master’s-level students, PhD and MPH programs have significant differences in focus and objectives, and programs for doctoral students are not geared toward the needs of practitioners.

### Conceptual framework for curriculum design

Our literature review of public health thought leaders indicated that future training for practitioners requires not only skills on how to implement well, but also in the allied fields of systems science, quality improvement, and evaluation to support implementation and are vital to the achievement of Public Health 3.0 goals. This is because the systematic implementation of interventions is only one of the drivers of successful and sustainable outcomes. The effect of even a well-implemented intervention may weaken over time if the system is not strong enough to sustain it, and improvements to delivery processes may be necessary. In some cases, especially in low-resource environments, the system may not even exist and may need to be designed from scratch.

**Fig. 2:**
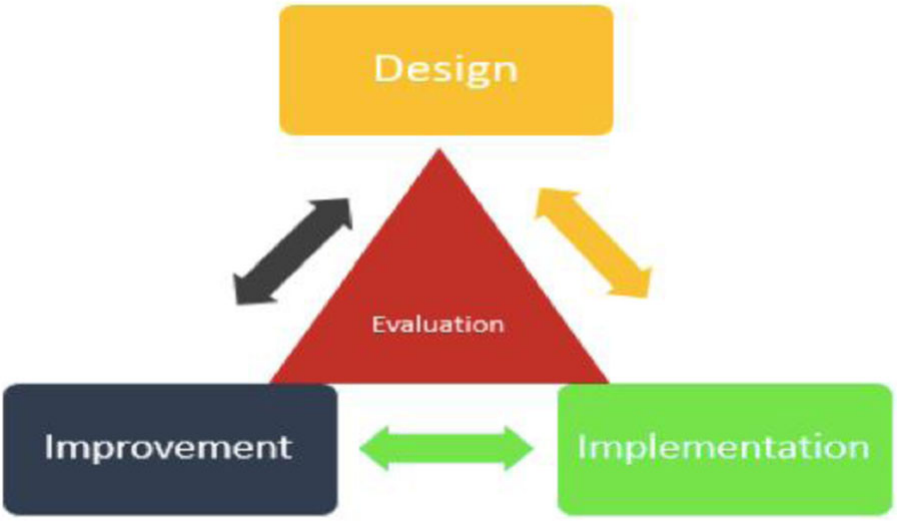
Design-focused implementation model (from Ramaswamy et al. 2018 [34])

Based on these considerations, we adapted a model termed *design-focused implementation* (Fig. 2) to guide our curriculum development. This model has been used to support the implementation of complex interventions in environments where the health system was weak [29]. The four components of the model (*design*, *implementation*, *improvement*, *and evaluation)* aligned well with the topics deemed important for training MPH students in implementation science [2, 3, 20].

### Course development

Our curriculum had four courses, each covering one component of the framework. The primary target audience for these courses was a cohort of MPH students enrolled in a newly created online MPH program. Three of these courses were developed from scratch for this program, and the fourth (Monitoring and Evaluation) was adapted to link its assignments with those of the other courses. The descriptions of the courses linked to each model component are shown in Table 1. Abridged course syllabi are provided in Additional file 1.

**Table 1:** MPH course titles and descriptions

Model component	Course name	Course description
Design	Designing Systems for Effective Implementation	Students apply design methods to create service delivery and support systems capable of achieving to successful health outcomes using a case study centered on the Ebola crisis in Liberia.
Implementation	Introduction to Implementation Research and Practice in Public Health	Students review core theories and methods in implementation research and applied practice employing a case study of the implementation of a program in India to address behaviors that are high-risk for HIV/AIDS.
Improvement	Applied Quality Improvement Methods for Healthcare and Public Health	Students learn quality improvement skills using a case study on improving clinic scheduling efficiencies.
Evaluation	Monitoring and Evaluation of Global Health Programs	Students learn concepts and tools for monitoring and evaluation of public health programs by revisiting the case study used in the Implementation course through an evaluation lens.

### Mapping course content to implementation research competencies

To assess the extent to which our curriculum and topics covered in the courses were in alignment with mainstream implementation research competencies, we compared the content of the four courses in our concentration against the competencies for dissemination and implementation training collaboratively developed by 124 researchers [30]. Even though these competencies were designed with researchers in mind, as indicated in Fig. 1, researchers and practitioners need a common base set of competencies to effectively complete the “virtuous cycle” [13]. Our mapping was done deductively by reviewing the topics covered in each course from the syllabus and determining whether there was a match between the topic and any of the implementation research competencies. This was done by two of the authors (RR and JM) through a collaborative dialogic process.

### Evaluation of training program

Two types of evaluation were conducted on the the training program. The first was a process evaluation as the courses were being taught aimed at real-time quality improvement. The directors of the online MPH program conducted synchronous online discussions to ascertain challenges faced by students in navigating the course content. These were conducted at least once, and sometimes twice during each semester. An interview guide was used to facilitate the conversation. Student participation was voluntary and sessions included at least eight students. Anonymous feedback from these sessions was passed on to the instructors and used to improve the courses in real time. No transcripts of these sessions were maintained, but changes to the courses based on student feedback were recorded in the learning management system.

The second evaluation employed the Kirkpatrick’s Model of Learning Evaluation (“reaction, learning, behavior, outcomes”) to evaluate initial student reaction, learning, and application of skills from the four courses [31]. These represent the first three levels of the Kirkpatrick model. Data for the evaluation utilized formal semester-end evaluations that are conducted for every course by the school of public health. These evaluations are standardized across all MPH courses and involve ten Likert scale questions and two qualitative questions. Most of the questions focus on course materials, instruction, and delivery, but one question, “*What will you take away from this course?*”, asks about the future use of learning from the course. We used de-identified answers to this question based on evaluation reports requested from the course instructors who received them at the end of each semester. In addition, student grades and documentation of practicums, projects, and master’s papers were used to assess how students learned and applied their learning.

## Results

### Findings from review of existing implementation science training offerings

We present the findings by each type of training offering described above. Given the rapid growth of interest in implementation science and the increasing number of institutions offering training, that there may be brand-new training programs for which online descriptions do not currently exist.

#### Researcher training programs not leading to a university degree, diploma, or certificate

The intensive workshops offered by the Implementation Research Institute (IRI) and the Training Institute on Dissemination and Implementation Research in Health (TIDIRH) are prime examples in this category [15, 32–34]. Based at Washington University in St. Louis, IRI has offered training in mental health implementation science to 2-year cohorts comprised of ten health researchers in annual cycles that commenced from 2010 to 2015 and again since 2016. In 2011, the IRI model inspired the U.S. Department of Veterans Affairs (VA) and collaborators to launch TIDIRH, which has targeted researchers who are interested in dissemination and implementation research across all areas of health. TIDIRH training is now structured around a combination of online, in-person, and group work, and participants are supported in seeking National Institutes of Health (NIH) and VA implementation-focused funding [15, 34]. This training model has recently been replicated in Ireland (TIDIRH-Ireland).

#### Stand-alone courses

Various institutions have begun offering stand-alone implementation science courses to various student audiences [35–37]. For example, the University of Michigan Medical School’s Health Infrastructures and Learning Systems program offers, to master’s and doctoral students, a two-semester sequence in “Implementation Science in Health” [35]. A second example is an annual, one-semester implementation science course for doctoral students, offered by the Department of Medical and Health Sciences at Sweden’s Linköping University, which uses a “problem-based learning” approach to train an average of 20 PhD students per year [37].

#### Graduate certificate, master’s, and doctoral programs

Graduate certificate programs in the USA for the most part are not offered by schools of public health [38–40]. For example, the Indiana University School of Medicine’s Graduate Certificate in Health Innovation and Implementation Science is geared toward health care professionals who want to learn how to enact change in the healthcare system [38]. The University of Nevada Las Vegas (UNLV) Certificate in Global Health and Implementation Science is offered by a school of public health. This certificate has a heavy focus on research and is mainly geared toward preparing students for the doctoral program at UNLV [40]. The University of California, San Francisco, offers a certificate program in implementation science through its school of medicine as part of its training in clinical research. This program is oriented toward researchers and favors those with prior master’s degrees in public health, clinical research, or epidemiology [39].

The only master’s level implementation science program in the USA is offered by the University of California San Francisco. This residential program offered through the school of medicine is geared toward clinical professionals (e.g., physicians) who want to focus on independent research or quality improvement careers [41].

There are several doctoral programs in implementation science in the USA [42–45] and three are offered by public health schools. Johns Hopkins University offers a DrPH program that focuses on training future public health professionals who want to pursue leadership positions [43]. This program requires that their students already have an MPH. Additionally, UNC offers a PhD program in Health Policy Management with a minor in Organization and Implementation Science. This residential program provides doctoral students with the education necessary to conduct implementation research [44]. The University of Washington offers a PhD in Global Health jointly with the school of medicine with an emphasis on implementation science and metrics [45]. While doctoral-level courses in implementation science may be available to MPH students, these are not typically oriented toward those seeking to apply the learning in practice.

### Findings on accessibility and fit of existing training programs to Public Health 3.0 needs

Our findings, presented as Additional file 2, suggest that there is not a single US-based graduate implementation science program focused on public health practitioners. The only US-based graduate certificate program (UNLV) taught in a school of public health positions students to enroll in the doctoral program. There are no master’s-level programs offered by schools of public health. The doctoral programs at Johns Hopkins, UNC and the University of Washington are intended to train professionals for careers in governmental and non-governmental organizations in addition to research institutions, but these are students who already have master’s degrees. Our findings clearly established a rationale for developing an implementation science program from scratch.

### Training program delivery structure

The courses were all delivered online with a mix of asynchronous and synchronous content. The course materials were available on the learning management system (Sakai), and the course content was broken down into 1 or 2-week modules. For each course, students were assigned three to four required readings each week and listened to a pre-recorded lecture. For most courses, each student was required to post one reflection to the readings and one clarifying question for each module. These questions were discussed in an online synchronous session conducted for each module. As indicated in Table 1, each course includes a progressive case study as a central pedagogical element.

To complete the case studies, students were divided into small groups of four to five students each, and were required to submit their work on progressive components of the case study for review at the end of each module, when they received feedback and guidance but were not graded on performance. At the end of the course, students were graded on a professional presentation of the complete case study, demonstrating the integrated use of the tools and concepts learned in the course in logical manner tools to address a real-life problem. Grades were based on the demonstrated ability of students not only to apply individual methods and tools, but also on their ability to integrate them.

### Enrollment statistics

As of spring semester 2018, we have offered a total of eleven sections of the four courses, attracting a total enrollment of 142, of whom 127 have been master’s-level students in the school of public health (the other enrollees are doctoral students or students in other schools who have taken one or more of these courses individually as electives). We have offered two sections of design (15 enrollees total); two of implementation (27); three of improvement (38); and four of evaluation (62).

### Findings from evaluation for quality improvement

The areas that were the most challenging to students were in translating abstract and conceptual literature into use in practice. The case studies were based on real projects, and students had difficulty selecting and applying implementation and evaluation frameworks post hoc to the case study scenarios, since the projects themselves had not used any implementation science concepts or tools. Students expressed frustration at the lack of guidance in the research literature on how to select and apply theories, models and frameworks, implementation strategies, or systems thinking approaches. Some students had difficulty in distinguishing between implementation and program planning, because some implementation frameworks overlap with planning frameworks. The course content and instruction were adapted in real time to address these concerns.

### Findings from end of semester evaluations

Student feedback evaluated across the first three levels of the Kirkpatrick model was positive. Since the students had just graduated, or were still in the program when the course evaluations were conducted, it was not possible to evaluate the fourth level of the Kirkpatrick model, which assesses external outcomes associated with training.

Student reactions (level 1) were assessed using answers to the question “What will you take away from this course?” Across all courses, we were able to obtain an evaluation from 38 students, and the summarized student responses are shown in Table 2. Broadly, qualitative student feedback in this regard was largely positive, with students reporting enthusiasm about their experiences in the various courses. This is not surprising because student issues and concerns were addressed as part of the QI process.

**Table 2:** Selected student comments from course evaluations, Summer 2015 through Fall 2017

Course	Representative student feedback
Design	“Course is invaluable especially because it is shaped around real-world situations.”
	“I most liked the exploration of agent-based systems”
	“I enjoyed the use of the (Ebola) case study across the full course”
	“[I found] NetLogo to be an excellent tool for representing and visualizing systems, including their emergent behavior”
	“[Will take away] a better understanding of...unique ways to solve public health [challenges] using tools that are not necessarily specific to public health”
	“[Will take away] a broad understanding of systems thinking and design thinking”
	“[Have learned how] to approach public health problems from a systems-thinking lens [and] how to use nontraditional methods of inquiry (design thinking tools) to gain a deeper understanding”
Implementation	“Useful resources, tools, and skills” for “application of implementation science concepts to global health”
	“[Acquired] basic skills need to apply [implementation concepts] to real-life situations”
Improvement	“A very deep dive into quality improvement techniques and applications”
	“I really enjoyed the more specific, practical way of approaching problems systematically. [We] spend so much time in other courses focusing on generating robust evidence for interventions that much of the practicality is left behind. This course complemented my other coursework very well.”
	“I will take away specific tools including the Driver Diagram, Swim Lane, PDSA cycle, and many others that I am already implementing in my professional work.”
	“The focus of this course on quality improvement added to my enthusiasm as implementation research and quality improvement. I have already used many of the tools and technique we have discussed in my work.”
	“[This course was] extremely practical and allows you to build real skills. Considering all other courses during my MPH, this class has been one of the best uses of my time.”
Evaluation	Course was “the most professionally applicable for me...I truly have a much better grasp of how to design and implement an evaluation. I am very pleased with how much context, examples, and explanation we got not just on the different types of evaluation, but when and where you’d use them, and how constraints of resources may affect that plan in real life.”
	“[S]ome of the most directly applicable coursework I have had for a long time”

Student learning (level 2) was assessed from grade reports of all offerings of the courses up to the Spring 2018 semester. All students who have completed these courses either received a grade of “entirely satisfactory graduate performance (a grade of P)” or “clear excellence (a grade of H)”, the two highest grades in the UNC grading system. Aggregated grade distributions across all offerings of a given course show that the proportions of students receiving H grades in a given course ranged from 15% (implementation) to 61% (improvement).

The degree to which students applied what they have learned (level 3) was supported by the fact that at least eight students embarked on practicums, master’s papers, and other learning products explicitly linked to the applied implementation science curriculum. These projects involved content from all the courses, demonstrating the importance of the interdisciplinary content to address real-life public health problems. Examples include practicums in the form of quality improvement studies of, respectively, wait times at a pediatric pulmonary clinic and patient safety in hospital contexts; master’s papers evaluating rural drinking water systems in low-resource settings and applying design methods to improve health care access among refugee populations; and an external evaluation project to gauge readiness for the introduction of a quality improvement program in maternal clinics in India.

## Discussion

By deliberately adding systems and design thinking and quality improvement courses to a curriculum on implementation science, and by emphasizing the interrelationships between the fields through the course instruction and the case studies, the UNC implementation science program looks different from the some of the other programs in our review. However, this does not mean that the students from the program are unable to be “mainstream” implementation scientists, as defined by the implementation research competencies described by Padek et al. [30]. The Padek study identified 43 competencies, categorized into beginner (*n* = 11), intermediate (*n* = 27), and advanced (*n* = 5). One hundred percent of the basic competencies, 71% of the intermediate competencies, and 0% of the advanced competencies are covered by at least one of our four courses. This is understandable in that the competencies were developed to “guide training in D&I research” whereas our focus is on building practice-based competencies. The details are shown in Additional file 3.

 Our curriculum reflects an emerging viewpoint that implementation science is part of a broader discipline encompassing multiple fields. The previously described NIH-convened panel acknowledged that dissemination and implementation research is its own distinct area of “health services, delivery system science, and system redesign” [17]. The panel further recognized that while training in implementation science should enable trainees to employ the methods in the field, it should also equip them to draw on methods from related research areas such as systems science, industry and engineering, safety, and improvement science (including quality improvement and healthcare improvement) [17].

Others have also acknowledged this need for implementation scientists to be able to draw from a variety of aligned disciplines. Northridge and Metcalf and Burke et al. argue that successful implementation cannot take place without acknowledging complications that occur in real-world situations and that the tools of systems science are needed to represent and model this complexity [46, 47]. Ogden and Fixsen and Meyers, Durlak, and Wandersman situate Continuous Quality Improvement methods such as Plan-Do-Study-Act (PDSA) within implementation science frameworks as an important tool for iterative learning and adaptation [48, 49]. Balasubramanian et al. and Patton describe the need for implementers to use evaluation methods such as developmental and learning evaluation to capture unexpected context-specific outcomes that may emerge as complex interventions are implemented in complex systems [50–52].

Our map of course content to the competencies identified by Padek et al. showed that all four courses mapped to these competencies were distributed across all the courses in our curriculum. Of the 29 competencies covered by at least one course, the implementation science course addressed 14 (48%); the evaluation course addressed 10 (34%); the systems course covered 7 (24%); and the improvement course met 5 (17%). A given competency could be addressed by more than one course. This lends credence to the validity of our interdisciplinary approach.

However, the diversity of our curriculum and our focus on endowing practitioners with usable yet rigorous skills has also brought to light the continuing gap that exists between the research literature and the needs of practitioners. Tools and approaches from systems thinking, design thinking, improvement science, and implementation science can all assist in tackling complex problems in public health, but many of these fields have evolved over decades and have their own language and concepts. The academic literature in any of these disciplines is theoretical and dense, and few guidelines exist about how to make the concepts in the literature accessible to practitioners. Despite our best attempts to develop an integrated curriculum and to use case studies to stimulate application, the students still struggled with how to “connect the dots” and make sense of the literature in a practical way. There is the need for faculty to act as translators to assist students with the connections across the disciplines and between research and practice. This is challenging in academic environments with highly specialized faculty. Moreover, before we can draw any generalizable conclusions about the effectiveness of this training, more research is needed. So far, we have observed our students’ demonstrated use of their learning in class projects, practicums, and papers with positive results. In future studies, we will need to evaluate whether this training of implementation practitioners results in better collaborations with researchers and if they are changing implementation and health outcomes. Specifically, we will need to research whether graduates from the program engage in a rigorous analysis of the system in which the interventions need to be delivered, whether they select appropriate system design activities if needed, whether they utilize theory-driven implementation strategies, and whether they proactively document the rationale for any adaptations to the interventions themselves or to the strategies.

## Conclusion

As we have been reminded by Joseph Durlak, a leading implementation scientist, “[W]hen it comes to implementation, what is worth doing, is worth doing well” [53]. If the next generation of public health practitioners is to succeed in closing the research-to-practice gap and drive transformative gains in public health, they must be equipped with a comprehensive skill set in implementation practice. Our training program provides this skill set and evaluations have demonstrated that it is well received and that students find the methods and tools that they learn in the course to be useful. Our initial assessments lead us to be optimistic that our applied implementation science training program prepares MPH students who represent the backbone of the future Public Health 3.0 workforce for success as public health implementers in the diverse, complex contexts where change at scale must be achieved. This allows us to cautiously promote this curriculum and training program to other schools of public health and possibly students from other disciplines such as social work or education who are also likely to have a need to implement evidence-based interventions in complex domestic and global settings. The key innovations that are worth considering by other programs are the integration of other disciplines such as systems thinking and improvement science into the implementation science curriculum and the use of case studies to illustrate the connection between these disciplines. This approach could serve to bridge the research-practice gap in a field that was specifically created to do so.

## Additional files

Additional file 1: Abridged course syllabi. (PDF 356 kb)

Additional file 2: Summary of Academic Implementation Science Training Programs. (XLSX 13 kb)

Additional file 3: Alignment of four courses with D&I competencies. (Padek, 2015) (XLSX 14 kb)

## Abbreviations

D&I: Dissemination and implementation
IRI: Implementation Research Institute
MPH: Master of Public Health
NIH: National Institutes of Health
SDG: Sustainable Development Goals
TIDIRH: Training Institute on Dissemination and Implementation Research in Health
VA: The U.S. Department of Veterans Affairs
