# Supplementary material for: Building capacity for Public Health 3.0: introducing implementation science into an MPH curriculum
Source: Implement Sci. 2019 Feb 28;14:18. doi: 10.1186/s13012-019-0866-6 (PMC6396520; doi:10.1186/s13012-019-0866-6)
Supplement: Supplementary file 1 — Abridged course syllabi. (PDF 356 kb) [file 13012_2019_866_MOESM1_ESM.pdf]

Designing Public Health Systems

Credit Hours: 3 hours

COURSE OVERVIEW

This course provides current and future public health leaders with an interdisciplinary toolkit (drawing on approaches and tools from engineering, management and the social sciences) to conceptualize, analyze and design effective public health and healthcare delivery systems for successful implementation. The emphasis is on creating new solutions to address systems challenges rather than on improving the performance of existing systems. Key topics include methods for representing and analyzing systems (using soft systems and systems dynamics approaches), tools for understanding patient and community experience and contexts (using experience based co-design tools such as empathy maps and journey maps), translation of needs to system requirement (using approaches such as quality function deployment), system design (using value stream mapping and service blueprinting methods) and systems analysis (using simulation and failure mode analysis). The class uses a progressive case study approach to balance the creative with the concrete, teaching a blend of adaptive and technical skills that both critically important to public health leaders.

COMPETENCIES AND LEARNING OBJECTIVES

This course is designed to help you achieve the following competencies and develop the following course learning objectives:

UNC-CH Leadership In Practice Concentration Specific Competency

Design transformational systems and innovative approaches to ensure effective public health practice.

Course Specific Learning Objectives

- Understand the role of systems in affecting healthcare and public health performance
- Use the soft system approach to address messy problems
- Identify complex systems and their characteristics
- Map complex systems using causal flow diagrams
- Define approaches for designing systems
- Apply methods to find patient and community needs
- Develop design requirements from needs
- Design and prototype service delivery processes and systems
- Assess and mitigate risks in design

COURSE PREREQUISITES

There is no prerequisite for PUBH 718.

ASSIGNMENTS AND EVALUATION

This course will include individual assignments, graded team assignments of a case study and discussions, peer evaluations and a semester paper. Brief summary of the assignments are provided below.

Component	Points
○ Individual posts (reflective post and question)	Not individually graded but will count towards participation grade
○ Module assignment (group or individual) – points vary by complexity	**Total 300 points**
○ Module 1	15
○ Module 2	15
○ Module 3	30
○ Module 4	15
○ Module 5	30
○ Module 6	15
○ Module 7	30
○ Module 8	15
○ Module 9	15
○ Module 10	30
○ Module 11	30
○ Module 12	15
○ Module 13	30
○ Module 14	15
○ Semester project and presentation (graded at the end of the semester) – instructor grade	**100 points**
○ Semester project and presentation (graded at the end of the semester) – peer grade	**50 points**
○ Peer participation evaluation	**25 points**
○ Instructor participation evaluation	**25 points**
**TOTAL**	**500 points**

Assignments

The case study assignment and the individual semester paper are both designed to assess students' acquisition of the concentration competency "*Design transformational systems and innovative approaches to ensure effective public health practice*" . The case study requires students to progressively use systems thinking and design thinking principles to redesign the system to improve outcomes for a complex public health problem. The individual semester paper evaluates students' ability to use the systems and design thinking skills they have learned to design innovative approaches to address a complex public health problem of their choosing.

Quizzes and Exams

There is no quiz or exam in this course.

Grading Notes

All members of a team will receive the same base grade for team assignments and, if required, adjusted down based on participation and punctuality on team assignments. Students will received individual grades for the individual assignment and for the semester paper.

Final course grades will be determined using the following grading scale:

**H**	≥ 90/100	**High Pass**: Clear Excellence
**P**	70 - 89	**Pass**: Entirely Satisfactory Graduate Work
**L**	60 – 69	**Low Pass**: Inadequate Graduate Work
**F**	< 60	**Fail**

SEMESTER-AT-A-GLANCE

Module/Unit	Topic	Assignment Due
**SECTION 1: COURSE INTRODUCTION**		
**Module 1**:	Contexts and Complexity: An Introduction to Systems	Assignment 1 (15 points)
**SECTION 2: DESCRIBING AND ANALYZING SYSTEMS**		
**Module 2**:	Systems Thinking Approaches	Assignment 2 (15 points) –
**Module 3**:	Soft Systems Methodology in Practice	Assignment 3 (30 points) –<br>**Potential topic for semester project**
**Module 4**:	Other Systems Approaches – Systems Dynamics and Complex Systems	Assignment 4 (15 points)
**Module 5**:	Systems Dynamics in Practice	Assignment 5 (30 points)
**Module 6**:	Modeling System Dynamics	Assignment 6 (15 points)
**Module 7**:	Simulation Models in Practice	Assignment 7 (30 points)<br>**Semester Project: System Description**
**Module 8:**	Introduction to Design Thinking	Assignment 8 (15 points)
**Module 9**:	Co-design: Collaborative Shaping of the Design Problem	Assignment 9 (15 points)

**Module 10**:	Eliciting User Needs	Assignment 10 (30 points)
**Module 11**:	Translating Needs into Performance Requirements	Assignment 11 (30 points)
**Module 12**:	Generating Transformation Ideas	Assignment 12 (15 points)
**Module 13**	Designing the System	Assignment 13 (30 points)
**SECTION 3: ANALYZING THE DESIGN**		
**Module 14**:	Prototyping and Testing	Assignment 14 (15 points)<br><br>**Semester Project: System Design**
**Project Presentation**		**(150 points)**

Introduction to Implementation Research and Practice in Healthcare and Public Health

(Credit Hours: 3)

Course Description: Internationally, there is a substantial gap between the development of innovations in public health and their delivery in routine practice. Implementation research and practice have emerged as a means of addressing that gap. Implementation research is defined as "the scientific study of methods to promote the systematic uptake of research findings and other evidence-based practices" to improve the quality of service delivery in routine care settings (Eccles & Mittman, 2006). It includes the study of influences on professional and organizational behavior that impact implementation effectiveness. This is one of the four courses in the Global Online MPH program's Implementation Science concentration. It will provide an overview of the core theories and methods in implementation research and practice and will cover implementation determinants and strategies at the intervention, individual, organizational, and policy levels. Through individual and group assignments, students will have numerous opportunities to apply principles to their own areas of interest and to implementation problems in global public health. In addition to course readings and individual and group assignments, regular online synchronous discussion sections will be scheduled to facilitate learning. At the conclusion of this course, students will be able to: 1) identify implementation gaps in a wide-range of US and global health service settings; 2) describe and apply core concepts of implementation research and practice; 3) use theories and frameworks to inform implementation research and practice; 4) assess multi-level barriers and facilitators (i.e., determinants) of implementation effectiveness; 5) apply strategies to implement, sustain, and scale-up effective practices; and 6) align their academic and applied work to priorities within the field of implementation science.

Summary Outline and Calendar

Module/Week:	Topic:	Assignment:
**Module 1**		
**W1:**	Introduction to Implementation Research and Practice	Introductory Post and Responses (Due ##)
**Module 2**		
**W2:**	Guiding Conceptual Frameworks and Theories	Ind. Paper 1 (Due ##)
**W3:**		Post 2 (Due ## and Responses (Due 1##)
**Module 3**		
**W4:**	Overview of Implementation Strategies; Intervention-Focused Determinants and Strategies	Ind. Paper 2 (Due ##)
**W5:**		Post 3 (Due ##) and Responses (Due ##)
**Module 4**		
**W6:**	Assessing Individual-Level Determinants; Individual-level Implementation Strategies	Group Paper 1 (Due ##)
**W7:**		Post 4 (Due ##) and Responses (Due ##)
**Module 5**		
**W8:**	Assessing Organizational-Level Determinants; Organizational-Level Implementation Strategies (Part 1)	Post 5 (Due ##) and Responses (Due ##)
**W9:**		Group Paper 2 (Due ##)
**Spring Break (3/9-315/2019)**		
**Module 6**		
**W10:**	Organizational-Level Implementation Strategies (Part 2)	Post 6 (Due ##) and Responses (Due ##)
**W11:**		Group Paper 3 (Due ##)
**Module 7**		
**W12:**	Assessing and Changing the Enabling Environment; Multi-Level Determinants and Implementation Strategies	Post 7 (Due ##) and Responses (Due ##)
**W13:**		None (Work on Final Assignments)
**Module 8**		
**W14:** 4	Sustainability and Scale-Up; Future Directions in Implementation Research and Practice	None (Work on Final Assignments)
**W15:**		Final Imp. Plan AND Course Reflection Paper (Due ##)

NOTE: Assignments are due at 11:55 p.m. EST on the dates listed.

Course Materials: All the materials for this course will be posted on Sakai. The materials for each week's lesson (including introductory PowerPoint presentation(s), readings, online lectures/webinars, and assignment details) will typically be posted no later than the preceding Saturday.

Course Structure and Activities: This course will involve:

1) completing assigned reading and review of other course materials,
2) participating in in person and online discussion sessions,

3) contributing and responding to content on the course discussion board,
4) the completion of individual and group papers that will allow you to apply key concepts to a case study pertinent to global public health, and
5) a final reflection paper in which you apply the lessons from this course to your own area of interest.

***Readings and Other Materials*:** Required readings are all listed below, along with additional, optional readings, online lectures/webinars, and useful tools. I will provide context for the readings, and help you to focus in on the key points that I hope that you take away from the assigned readings and any optional materials. You are also encouraged to seek out articles in your area of interest to supplement course material. All readings and additional course materials will be posted on Sakai in the “Resources” section.

***Synchronous Online Discussion Sessions*:** Approximately every 2 weeks we will have an online synchronous discussion session that will last between 30-60 minutes. These sessions will be mandatory, and we will work as a class to schedule a time that works for the class. These sessions serve three primary purposes: First, they will provide me the opportunity to introduce new material and to draw connections between the readings, course materials, and resources. Second, they will allow us to learn from each other’s unique experiences and expertise as we strive to apply implementation-related concepts to different public health problems and contexts. Finally, it will give you a chance to ask any lingering questions or concerns that arise throughout the semester.

***Discussion Board Posts and Responses*:** One of the ways that we will engage with the material and with each other throughout the semester will be through the discussion board. While it is always an option to facilitate dialogue through the discussion board, you will be formally required to post and to interact with your classmates on 7 occasions as noted in the summary outline above.

***Individual and Group Papers*:** Individual and group papers will give you an opportunity to apply the content of this course. Except for the final group paper, these papers will all be relatively brief (1000 word limit). Individual papers 1 & 2 will focus on articulating the relevance of implementation research and practice and applying conceptual frameworks, respectively. The group papers will focus on the application of course content to a case study. Group papers 1-3 will provide a solid foundation for the final group paper, which will involve developing a comprehensive implementation plan for your group’s specific public health problem.

***Final Course Reflection Paper*:** You will be given detailed instructions for a final course reflection paper (1500 word limit) that will allow you to demonstrate your comprehension of key course concepts and your ability to articulate their relevance to your area of interest.

Grade Components:

**Assignments and Course Activities:**	**Points:**
Discussion Board Posts (7)	10
Individual Paper 1	10
Individual Paper 2	10
Group Paper 1	10
Group Paper 2	10
Group Paper 3	10
Final Implementation Plan	15
Final Course Reflection Paper	10
Participation	15
**Total**	**100**

Grading Scale*:

90-100: ***Honors*** indicates exceptional graduate-level work. Assignments must meet or exceed performance expectations, as defined for each assignment.

70-89: ***Pass*** indicates that assignments are acceptable with regard to both content and presentation, but contain one more deficiencies related to performance expectations.

65-69: ***Low pass*** indicates marginally acceptable graduate-level work. Assignments show some major deficiency with respect to content or presentation.

< 65: Fail indicates that the assignment does not meet an acceptable level for graduate-level work.

*These are guidelines, and are not meant to be absolute numbers.

Monitoring and Evaluation of Global Health Programs

(Credit Hours: 3)

Course Description

This course covers the fundamental concepts and tools for monitoring and evaluation of global public health programs. Basic concepts and practices in M&E will be covered such as formative research, stakeholder engagement, conceptual frameworks, data collection methods, indicator development, survey and focus group design, performance monitoring and designs for process, outcomes, impacts and efficiency.

PUBH 714 Summer 2017 Calendar				
**Dates**	**Topic**	**Quiz**	**Classwork**	**Small Group Project**
**Week 1: May 17-21**	**Intro to M&E**	Yes	Yes	N/A
**Week 2: May 22-28**	**Program Theory in Evaluation**	Yes	Yes	Assign to Small Groups
**Week 3: May 29-June 4**	**Developing Evaluation Questions with Stakeholders**	Yes	Yes	Start Project
**Week 4: June 5-11**	**Indicators for Measuring Performance**	Yes	Yes	Part #1 due (June 11)
**Week 5: June 12-18**	**Data Collection and Questionnaire Design**	Yes	Yes	
**Week 6: June 19-25**	**Sampling Approaches**	Yes	Yes	Part #2 due (June 25)
**Week 7: June 26- Jul 2**	**Monitoring Progress, Evaluating Processes**	No	Yes	
**Week 8: July 3- 9**	**Randomized Experiments**	Yes	No	Part #3 due (July 9)
**Week 9: Jul 10 – 16**	**Quasi/Non-experimental Designs**	Yes	Yes	
**Week 10: Jul 17-23**	**Mixed Methods Design**	Yes	No	Part #4 due July 20
**Week 11: July 24-Aug 1**	**GROUP WORK/EXAMS**	**FINAL EXAM July 31**	No	**FINAL DRAFT (+ Part 5) DUE July 28**

Grading Scheme

Grading components

Your final grade will be based on a combination of *one exam (20%), nine individual quizzes (20%)*, a *group project (40%)*, and *participation (20%)*.

Each module will have **a quiz** which is due at midnight on Sunday at the end of the module.

There is **one group project**, with intermediate deliverables throughout the semester, though intermediate deliverables will not be graded.

Some weeks you will be asked to contribute to a short **in-class discussion** which will not be graded but will contribute to your overall participation grade.

As a member of a team, you are expected to participate regularly and provide input into the group assignments. Participation does not only mean passively providing input – it also means reacting to what your peers have said and contributing to discussion online. Your participation grade will be based on faculty evaluation and on one peer assessment. At the end of the semester, you will have the opportunity to evaluate your group members on your perception of their level of participation and contribution to the group.

There will be a final exam, one week before the end of semester. It will be open book, open notes and take home.

Breakdown of components - The distribution of points will be as follows:

Individual quiz - 20%

Final exam: **20%**

Group Project Presentation and Report: 40%

Participation in discussion: (Instructor assessment and peer evaluation): **20%**

Applied Quality Improvement for Healthcare and Public Health

(Credit Hours: 3)

Course Overview:

This is a three-credit hour, online graduate-level, interdisciplinary course in which teams of students apply quality improvement methods to improve processes of delivery of public health services.

Students will work in teams and use a systematic quality improvement method (the Model for Improvement) on a real life case study to analyze performance, identify sources of variability in performance, and develop and test improvement solutions. The skills gained in this course are applicable to a wide variety of health care and public health situations in the US and globally.

Course Objectives:

Through this course(s), students will:

1. Become familiar with Quality Improvement (QI) methods, especially the Model for Improvement;
2. Develop skills in using the tools of QI to solve a real-life improvement problem
3. Develop generalizable insights about use of QI to facilitate local and global improvements in health care and public health;

Competencies:

The course is designed to support student attainment of the following Global Health Core Competencies (ASPPH 2011):

1. Assist host entity in assessing existing capacity;
2. Conduct a situation analysis across a range of cultural, economic, and health contexts;
3. Develop monitoring and evaluation frameworks to assess programs;
4. Exhibit interpersonal communication skills that demonstrate respect for other perspectives and cultures;
5. Develop strategies that strengthen community capabilities for overcoming barriers to health and well-being;
6. Design context-specific health interventions based upon situation analysis;
7. Design program work plans based on logic models;
8. Develop context-specific implementation strategies for scaling up best-practice intervention;
9. Apply scientific evidence throughout program planning, implementation, and evaluation;

In addition, the course is designed to support student knowledge in the following content areas for the common core of the MPH degree (ASPPH 2015):

- Systems thinking regarding the dynamic interactions among sectors, organizations, and actors with which public health professionals interact to achieve health improvements
- Concepts of project implementation and management, including planning, budgeting, human resources, assessment, and evaluation
- The cultural context of public health issues and respectful engagement with people of different cultures and socioeconomic strata
- Principles of effective functioning within and across organizations and as members of interdisciplinary and interprofessional teams

Course Requirements:

Four requirements will be the basis for assigning grades for this course:

1. Successful completion of online quizzes associated with each module.
2. Participation in the online group case study which builds progressively through each module.
3. A group report describing the problem, analysis, solution, implementation, results and recommendations based on the case study.
4. A final exam.

As a member of a team, you are expected to participate in the case study. Participation does not only mean passively providing input – it also means reacting to what your peers have said and contributing to discussion online. Your participation grade will be based on faculty evaluation and on **peer assessments.** At the end of the course, you will have the opportunity to evaluate your group members based on your perception of their level of participation and contribution to the group. We will use this feedback both for improving participation and for evaluation at the end of the course.

Grading:

The distribution of points for each course requirement is shown below:

Requirement	% of Grade	Points possible
1) Quizzes	15%	15
2) Case Study report and presentation	30%	40
3) Final exam	35%	35
4) Participation (reflection question and case study)	20%	20
Total	100%	100

The final report and presentation will be graded on the following dimensions:

- Logical, appropriate, evidence-based conclusions, analyses, and recommendations in both the presentation and paper (30 percent)
- Effectiveness of presentations (30 percent)
- Clearly written report with topics arranged logically. Well-designed tables and figures that convey relevant, important information (20 percent)
- Effective, appropriate application of course materials and other resources in the presentation and paper (20 percent)

Grading will be according to the following scheme:

Grade	Explanation
H	Clear Excellence
P	Entirely Satisfactory
L	Low Passing
F	Fail

Typically, H grades are given to those scoring 90% or above, P to scores of 70% and above and L to scores of 55% and above. These are guidelines, and are not meant to be absolute numbers.

A grade of H will indicate that you have gone beyond the expectations of the assignment and have produced an exceptional output. A P is completely acceptable and indicates that you met the expectations of the assignment. An L indicates that you have turned in passing performance, but that the effort is minimally acceptable.

Semester at a Glance

Module 1	Introduction to Principles of Improvement
Module 2	Beginning an Improvement Project
Module 3	Viewing Work as a Process
Module 4	Collecting Data for Improvement
Module 5	Analyzing Baseline Data for Patterns and Trends
Module 6	Generating and Evaluating Improvement Solutions
Module 7	Testing and Implementing Solutions
Module 8	Adapting and Sustaining Solutions
